# Evaluating the Impact of Retinal Vessel Segmentation Metrics on Retest Reliability in a Clinical Setting: A Comparative Analysis Using AutoMorph

**DOI:** 10.1167/iovs.65.13.24

**Published:** 2024-11-14

**Authors:** Samuel D. Giesser, Ferhat Turgut, Amr Saad, Jay R. Zoellin, Chiara Sommer, Yukun Zhou, Siegfried K. Wagner, Pearse A. Keane, Matthias Becker, Delia Cabrera DeBuc, Gábor Márk Somfai

**Affiliations:** 1 Department of Ophthalmology, Stadtspital Zürich, Zurich, Switzerland; 2 Spross Research Institute, Zurich, Switzerland; 3 Gutblick Research, Pfäffikon, Switzerland; 4 NIHR Biomedical Research Centre at Moorfields Eye Hospital NHS Foundation Trust, London, United Kingdom; 5 Institute of Ophthalmology, University College London, London, United Kingdom; 6 Department of Medical Physics and Biomedical Engineering, University College London, London, United Kingdom; 7 Department of Ophthalmology, University of Heidelberg, Heidelberg, Germany; 8 Bascom Palmer Eye Institute, Miller School of Medicine, University of Miami, Miami, Florida, United States; 9 iScreen 2 Prevent LLC, Miami, FL, United States; 10 Department of Ophthalmology, Semmelweis University, Budapest, Hungary

**Keywords:** machine learning, image analysis, retest reliability, oculomics

## Abstract

**Purpose:**

Current research on artificial intelligence–based fundus photography biomarkers has demonstrated inconsistent results. Consequently, we aimed to evaluate and predict the test–retest reliability of retinal parameters extracted from fundus photography.

**Methods:**

Two groups of patients were recruited for the study: an intervisit group (*n* = 28) to assess retest reliability over a period of 1 to 14 days and an intravisit group (*n* = 44) to evaluate retest reliability within a single session. Using AutoMorph, we generated test and retest vessel segmentation maps; measured segmentation map agreement via accuracy, sensitivity, F1 score and Jaccard index; and calculated 76 metrics from each fundus image. The retest reliability of each metric was analyzed in terms of the Spearman correlation coefficient, intraclass correlation coefficient (ICC), and relative percentage change. A linear model with the input variables contrast-to-noise-ratio and fractal dimension, chosen by a *P*-value–based backward selection process, was developed to predict the median percentage difference on retest per image based on image-quality metrics. This model was trained on the intravisit dataset and validated using the intervisit dataset.

**Results:**

In the intervisit group, retest reliability varied between Spearman correlation coefficients of 0.34 and 0.99, ICC values of 0.31 to 0.99, and mean absolute percentage differences of 0.96% to 223.67%. Similarly, in the intravisit group, the retest reliability ranged from Spearman correlation coefficients of 0.55 and 0.96, ICC values of 0.40 to 0.97, and mean percentage differences of 0.49% to 371.23%. Segmentation map accuracy between test and retest never dropped below 97%; the mean F1 scores were 0.85 for the intravisit dataset and 0.82 for the intervisit dataset. The best retest was achieved with disc-width regarding the Spearman correlation coefficient in both datasets. In terms of the Spearman correlation coefficient, the worst retests of the intervisit and intravisit groups were tortuosity density and artery tortuosity density, respectively. The intravisit group exhibited better retest reliability than the intervisit group (*P* < 0.001). Our linear model, with the two independent variables contrast-to-noise ratio and fractal dimension predicted the median retest reliability per image on its validation dataset, the intervisit group, with an *R*^2^ of 0.53 (*P* < 0.001).

**Conclusions:**

Our findings highlight a considerable volatility in the reliability of some retinal biomarkers. Improving retest could allow disease progression modeling in smaller datasets or an individualized treatment approach. Image quality is moderately predictive of retest reliability, and further work is warranted to understand the reasons behind our observations better and thus ensure consistent retest results.

The automated assessment of retinal fundus images presents the opportunity to generate cost-effective, easily accessible, non-invasive, and objective biomarkers. Some of these biomarkers have demonstrated associations with various medical conditions, such as the association of cup-to-disc-ratio with glaucoma,^1^ and retinovascular parameters have been associated with hypertension,^2^ increased risk of stroke,^3^ all-cause mortality,^4^ obstructive sleep apnea syndrome,^5^ and hypercholesterinemia.^6^

In recent years, multiple open-source automated fundus-photography image analysis programs, such as the Retina-based Microvascular Health Assessment System (RMHAS),^7^ Integrative Vessel Analysis (IVAN),^8^^,^^9^ Quartz,^9^ Singapore I Vessel Assessment (SIVA),^9^ AutoMorph,^10^ and the Vascular Assessment and Measurement Platform for Images of the Retina (VAMPIRE),^8^^,^^9^ have been developed and are actively used in clinical research.^10^^–^^14^ However, subsequent research has reported poor agreement on the measured metrics across segmentation algorithms; a comparative analysis between VAMPIRE and SIVA reported an intraclass correlation coefficient (ICC) range of 0.16 to 0.41 for all extracted retinal parameters,^15^ which makes their robustness questionable in cases of missing significance when applied for research.^16^^,^^17^ The low measurement agreement in the literature raises serious concerns about the retest reliability of these biomarkers which could be concealing existing correlations between the biomarkers and ocular disease. Conversely, an improved retest could uncover a much stronger correlation between examined biomarkers and disease progression. AutoMorph is a fully automated, open-source algorithm that comes with an integrated image-quality grading algorithm and vessel segmentation algorithm. AutoMorph provides a wide array of extracted retinal metrics based on the calculated segmentation maps. AutoMorph has been tested on external datasets and has achieved good segmentation consistency with ground truths across imaging devices, collection methods, and datasets. AutoMorph reached a binary vessel segmentation area under the receiver operating characteristic (AUC) curve of up to 0.98 on test datasets.^10^

Our study addressed two main research questions: First, we sought to identify fundus image metrics that exhibit satisfactory retest quality and can be effectively utilized in real-world clinical settings. Second, we aimed to determine which fundus image metrics would benefit most from using an improved segmentation algorithm, alternative and more stable calculation methods, or more standardized image-capturing techniques.

## Methods

### Data Acquisition

For this study, we recruited two cohorts of patients. The first group, the intravisit group (*n* = 44) had a mean age of 41.3 years (range, 20–70). This cohort had only one session in which macula-centered fundus images were taken twice in both eyes. No subject was given mydriatic eye drops. After entering the darkened room, we waited for 2 minutes for the eyes of the patient to adjust to the dark, then took two photographs, one of each eye. After a 5-minute break, a second set of photographs was taken of each eye. Subjects removed their chin from the rest but did not leave the darkened room. The group consisted of a cross-section of staff, patients, and visitors to Stadtspital Zurich Triemli, recruited over 2 days. We excluded people with ophthalmic surgery in the past 6 months or anyone with any history of ophthalmic disease.

The second group, the intervisit group (*n* = 28) had a mean age of 54.8 years (range, 45–64). Fundus photography of each eye was taken at two different sessions over the course of 1 to 14 days. Fundus photography was performed after entering a darkened room and waiting 2 minutes. No subject was administered mydriatic eye drops. The group consisted of patients with phakic or pseudophakic eyes; in the case of pseudophakia, the preoperative spherical equivalent ranged from −3.0 to +3.0 diopters. All patients had a best-corrected visual acuity of 0.6 or better, measured with a Snellen chart at a distance of 5 meters. Patients were excluded if they had amblyopia, cataract, or ophthalmic surgery in the past 6 months; a history of retinal surgery; macular pathologies involving the fovea; intraocular pressure greater than 20 mmHg; or progressive optic neuropathy. After a participant provided informed consent, foveal-centered, non-mydriatic 45° fundus images were taken using a VISUCAM Pro NM camera (Carl Zeiss Meditec, Jena, Germany). Inter- and intravisit groups were independent; there was no subject present in both groups.

### Segmentation and Metric Calculation

**Figure 1.**
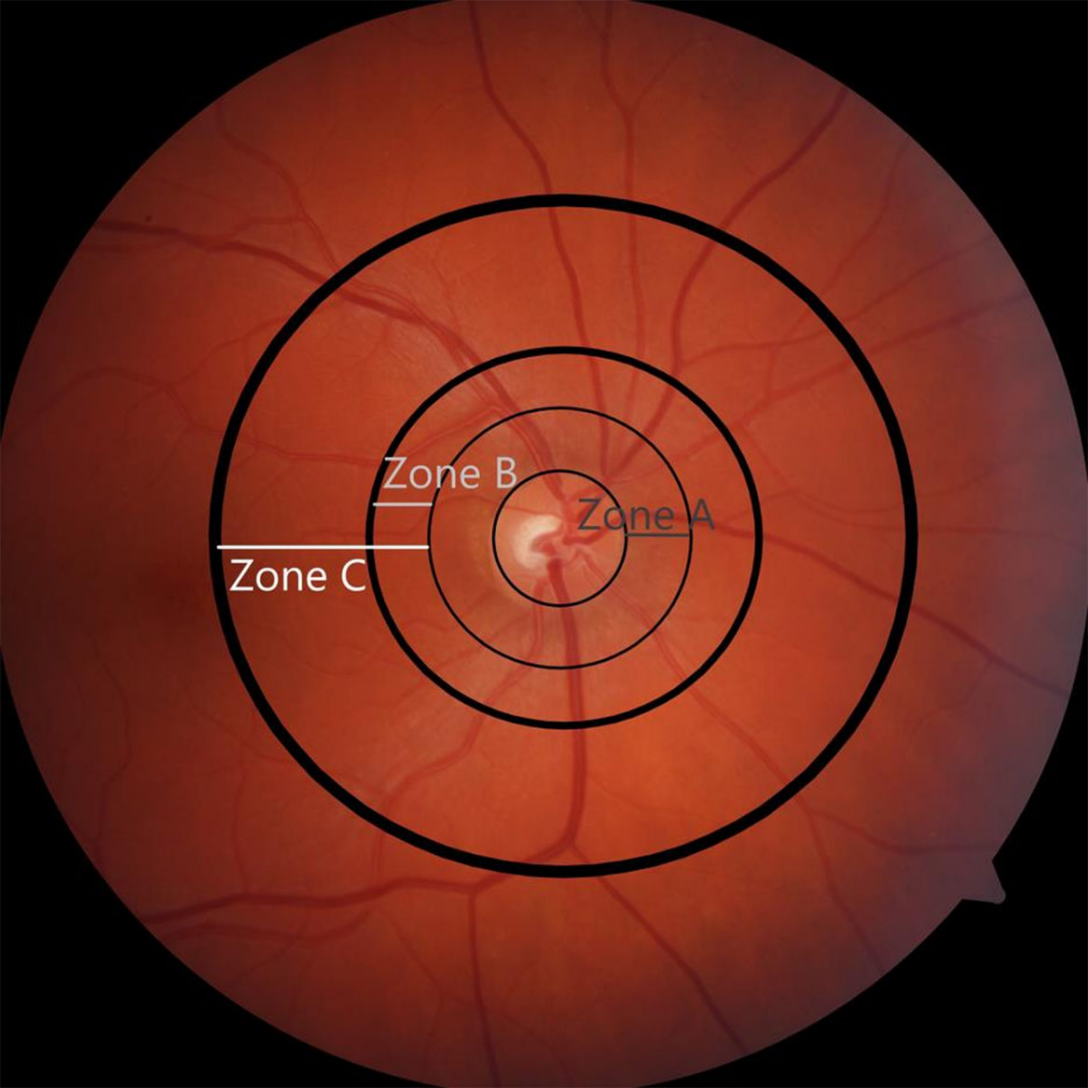
Diagram illustrating the different zones of a fundus photograph. AutoMorph reports on the entire image, Zone B (the annulus 0.5–1 optic disc diameter from the disc margin), and Zone C (the annulus 0.5–2 optic disc diameter from the disc margin).

AutoMorph receives a fundus image as input and examines the image quality of the fundus photograph. To do this, the image quality algorithm combines the outputs from eight distinct quality-grading models, each trained on different data. Subsequently, a confidence analysis is performed across the results of these eight different models. Images with low confidence in the classification, as well as those images with a high discrepancy in the image quality grading of the models, are flagged as ungradable.^10^ These images are discarded from subsequent analysis. While poor-quality images are discarded, for good-quality images a segmentation map of the vascular tree and the optic nerve head is created from the fundus image. Each vessel is classified as an artery or vein. In our study, we calculated 76 variables per fundus image based on the generated segmentation maps. AutoMorph provides most metrics for different zones of the retina. These zones are the entire macula observable of the image, Zone B (the annulus 0.5–1 optic disc diameter from the disc margin) and Zone C (the annulus 0.5–2 optic disc diameter from the disc margin) (Fig. 1). Table 1 gives an overview of the calculated metrics; a complete list is provided in Supplementary Tables S1 and S2.

**Table 1.** Overview of Recorded Metrics

	Tortuosity (Distance, Squared Curvature, Density)	Caliber (CRAE, CRVE, AVR)	Other (Fractal Dimension, Vessel Perfusion Density, Average Width)	Disc/Cup (Height, Width, CDR Horizontal, CDR Vertical)
Artery	Macula, Zones B and C	Macula, Zones B and C	Macula, Zones B and C	No
Vein	Macula, Zones B and C	Macula, Zones B and C	Macula, Zones B and C	No
Arteries and veins	Macula, Zones B and C	No	Macula, Zones B and C	No
Optic disc	No	No	No	Yes

AVR, arteriolar-venular ratio; CDR, cup-to-disc ratio; CRAE, central retinal arteriolar equivalent; CRVE, central retinal artery vein equivalent.

For both inter- and intravisit groups, we report on the segmentation map agreement between test and retest. To quantify segmentation map agreement, we used the metrics of accuracy, sensitivity, Jaccard index, and F1 score. In the reporting of each of these scores, we provide the minimum, mean, and maximum of the respective dataset.

We measured retest reliability with the Spearman correlation coefficient, two-way agreement, single-rater ICC, and percentage difference on retest. The overall retest quality of metrics is reported using cumulative distribution plots. We provide Bland–Altman plots for the four best and worst metrics of each group. Using signed Wilcoxon rank-sum tests, we tested for statistical differences between the metrics for the entire macula and Zones B and C. A complete ranking of all metrics with regard to Spearman correlation, ICC, and mean percentage difference between test and retest may be found in Supplementary Tables S1 and S2.

We assessed the impact of age and gender on retest reliability. To do this, we calculated the median relative difference between test and retest for each patient across all extracted metrics. We then performed a linear regression analysis to evaluate the correlation between age and retest reliability, and we used the Wilcoxon rank-sum test to determine if gender differences significantly influenced retest reliability.

### Image Quality Assessment

To assess the impact of image quality on retest reliability, we measured the image quality of every fundus image via the following:

• Fractal dimension (via box-counting method)^18^
• Sharpness (via gray Laplacian image transformation)^19^
• Edge acutance (via open-source python canny edge)^20^
• Contrast (standard-deviation–based contrast)^21^
• Colorfulness index^22^
• Contrast-to-noise ratio^23^
• Image entropy^24^

We tested each of these metrics for its predictive value on the median percentage difference on retest per image. For the intravisit dataset, we fit a generalized linear model consisting of the Box–Cox-transformed, statistically significant, most predictive, and non-collinear image quality metrics to predict the median percent differences on retest. The model used all statistically significant image quality variables and then performed a *P*-value–based backward selection until we arrived at a variance inflation factor (VIF) lower than 5. We validated this linear model on the intervisit group. All statistical analyses were performed in R (R Foundation for Statistical Computing, Vienna, Austria), and imaging analyses were performed in Python. The level of significance was set at 5%. This study adhered to the tenets of the Declaration of Helsinki, and all procedures involving human participants were conducted in accordance with ethical standards and approved by the ethics committee.

## Results

### Image Quality Grading

**Figure 2.**
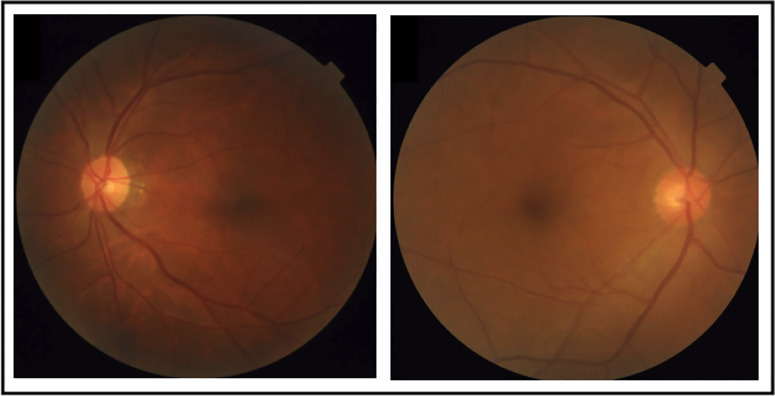
Examples of the automated grading algorithm from the FIVES open-source dataset. The *left side* shows an image graded as bad quality and thus discarded from further analysis, most likely due to the shadow in the outer parts of the fundus image. The *right side* shows an image that was barely graded as good quality and was included in analysis.^34^

The intervisit group had a total of 112 fundus images taken. After removing poor-quality images identified via the automated grading algorithm, a total of 70 fundus images were left available in a test–-retest setting for final analysis. Likewise, the intravisit group had a total of 176 fundus images taken, and, following image quality analysis, we were left with a total of 148 fundus images for final analysis. Figure 2 shows two edge cases that were graded good and poor quality by the grading algorithm.

### Segmentation Map Agreement for the Intervisit Group

Table 2 shows the segmentation map agreement between test and retest for the intervisit group.

**Table 2.** Segmentation Map Agreement Measured by Accuracy, Sensitivity, Jaccard Index, and F1 Score

	Intervisit Dataset		
Metric	Minimum	Mean	Maximum
Accuracy	97%	98%	98%
Sensitivity	0.81	0.83	0.86
Jaccard index	0.67	0.70	0.76
F1 score	0.79	0.82	0.86

### Retest Reliability Quantification for the Intervisit Group

**Figure 3.**
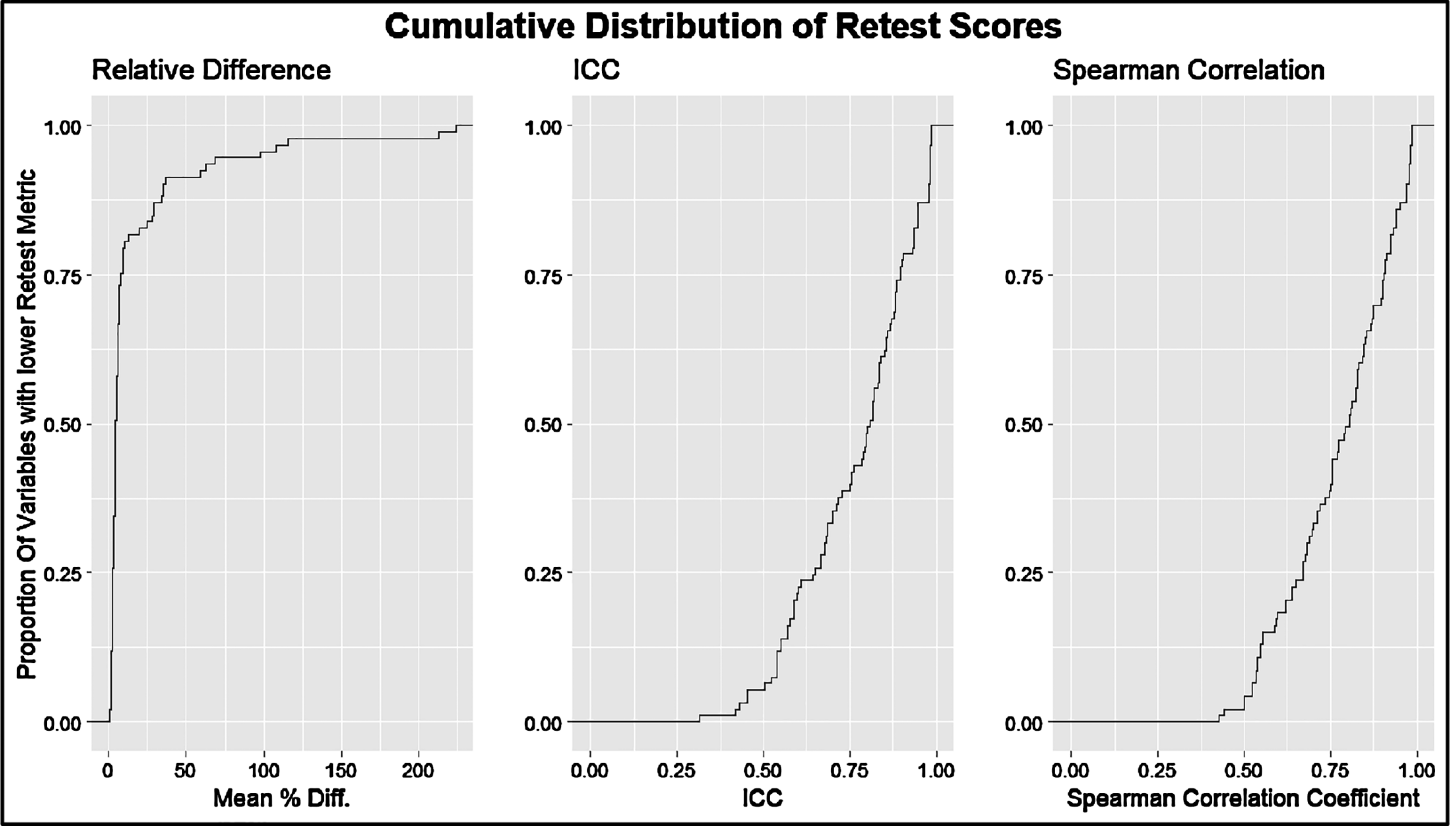
Cumulative distribution curves of retest quality metrics for the intervisit group. The *y*-axis represents the proportion of measured variables with lower retest. The *x*-axis from left to right shows the mean percentage difference between the test and retest metrics of two-way, single-unit agreement ICC and Pearson correlation coefficient.

**Figure 4.**
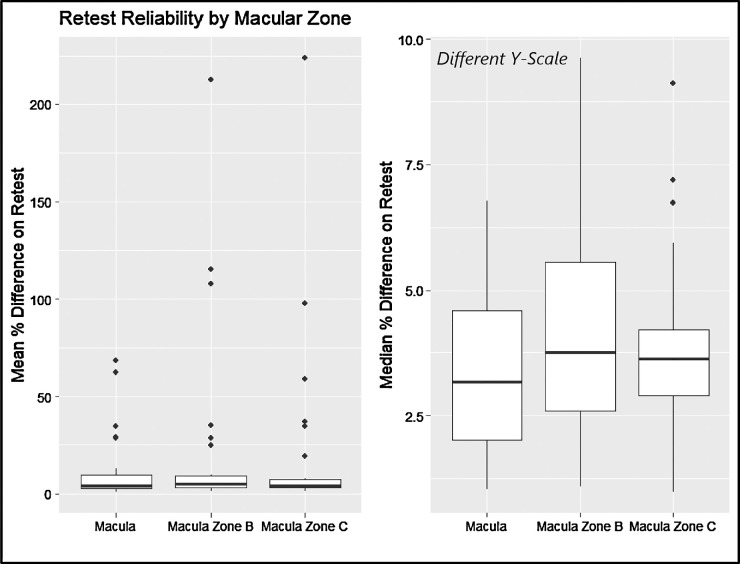
Mean (*left*) and median (*right*) percentage difference by zone for the intervisit group. Note the different *y*-scales of the box plots.

**Figure 5.**
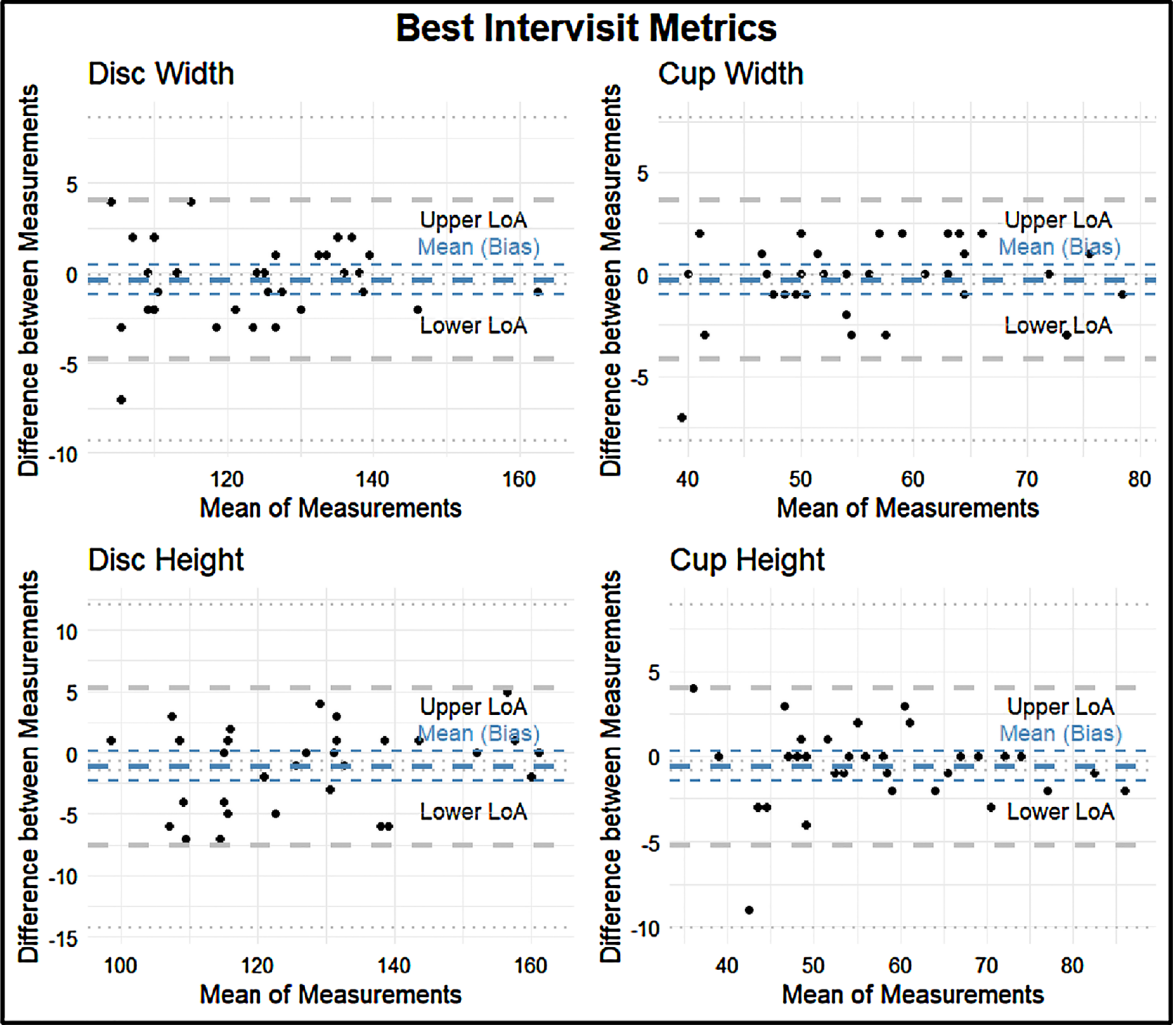
Bland–Altman plots for the four metrics with the highest retest reliability as assessed by Spearman correlation: disc width, cup width, disc height, and cup height. The *thick blue* *line* indicates the means (bias) of the measurements. The *thick* *gray* *lines* indicate the upper and lower limits of agreement (LoA). The *thin* *lines* indicate the 95% CIs for the means (bias) and LoA.

**Figure 6.**
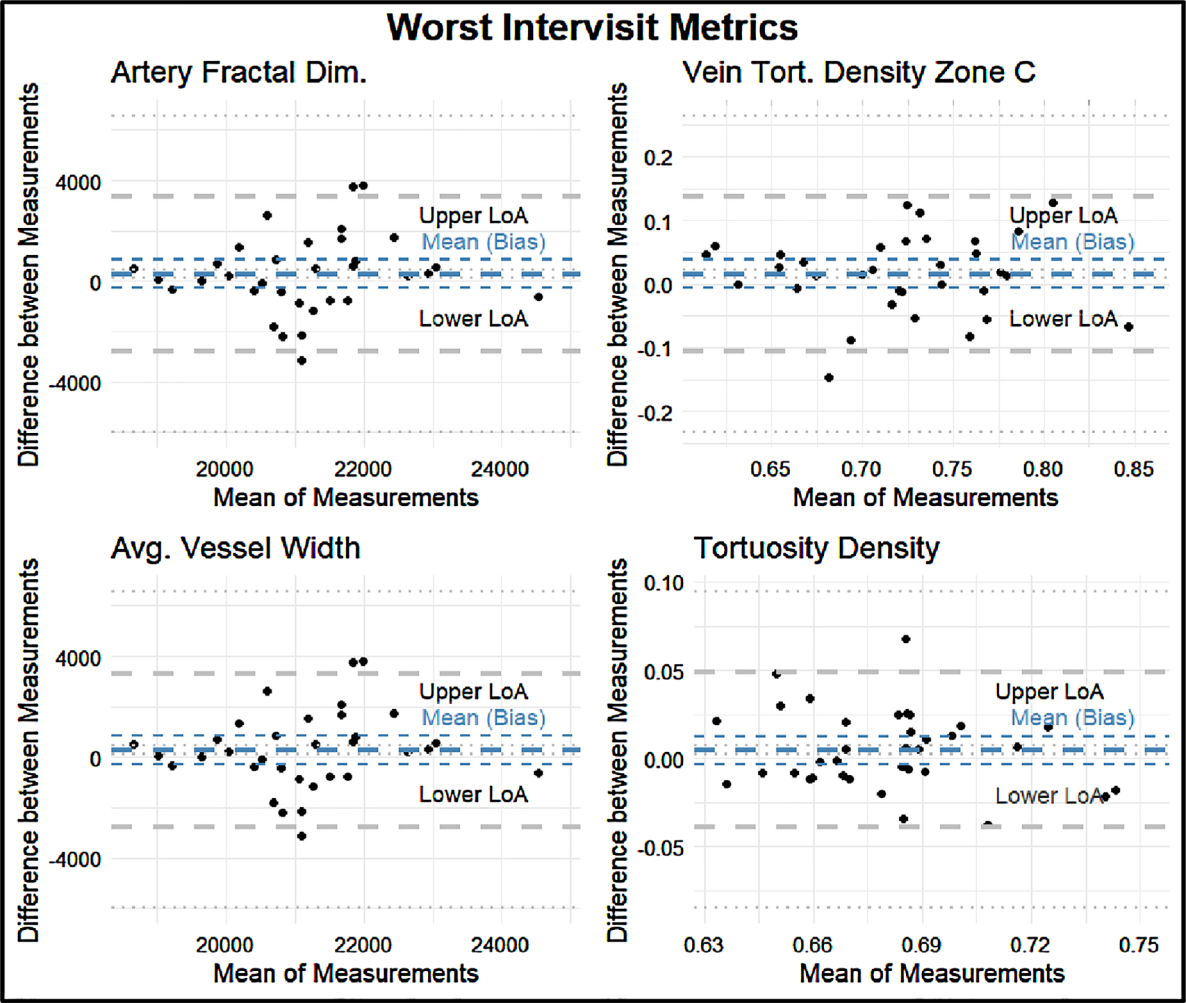
Bland–Altman plots for the four metrics with the lowest retest reliability as assessed by Spearman correlation: artery fractal dimension, vein tortuosity density in Zone C, average vessel width, and tortuosity density. The *thick blue line* indicates the means (bias) of the measurements. The *thick gray lines* indicate the upper and lower LoA. The *thin lines* indicate the 95% CIs for the means (bias) and LoA.

The intervisit group had a mean percent difference range of 0.96% to 223.67%, an ICC range of 0.31 to 0.99, and a Spearman correlation coefficient range of 0.34 to 0.99 (Fig. 3). For the intervisit group, the mean percent differences on retest ranged from 0.96% to 68.51% in the entire macula, from 3.43% to 212.80% in Zone B, and from 3.07% to 223.67% in Zone C. The median retest ranged from 0.97% to 6.95% for the entire macula, from 1.01% to 9.54% for Zone B, and from 0.91% to 9.07% for Zone C (Fig. 4). The four variables with the best retest in the intervisit group with regard to the Spearman retest were disc width, cup width, disc height, and cup height. The four worst were artery fractal dimension, vein tortuosity density of zone C, average vessel width, and tortuosity density (Figs. 5, 6; Table 3).

**Table 3.** Lower LoA, Mean LoA, and Upper LoA for each Bland–Altman Plot With a Precision of Two Significant Figures

	LoA		
Metric	Lower	Mean	Upper
Best intervisit metrics			
Disc width	−4.9	−0.12	4.7
Cup width	−4.4	0.03	4.4
Disc height	−6.9	−0.9	5.1
Cup height	−5.0	−0.15	4.8
Worst intervisit metrics			
Artery fractal dimension	−3600	250	3900
Vein tortuosity density zone C	−0.10	0.021	0.14
Average vessel width	−3500	240	3800
Tortuosity density	−0.050	0.00	0.050

### Segmentation Map Agreement for the Intravisit Group

Table 4 shows the segmentation map agreement between test and retest for the intravisit group.

**Table 4.** Segmentation Map Agreement Measured by Accuracy, Sensitivity, Jaccard Index, and F1 Score

	Intravisit Dataset		
Metric	Minimum	Mean	Maximum
Accuracy	97%	98%	98%
Sensitivity	0.84	0.85	0.88
Jaccard index	0.72	0.74	0.78
F1 score	0.84	0.85	0.88

### Retest Reliability Quantification for the Intravisit Group

**Figure 7.**
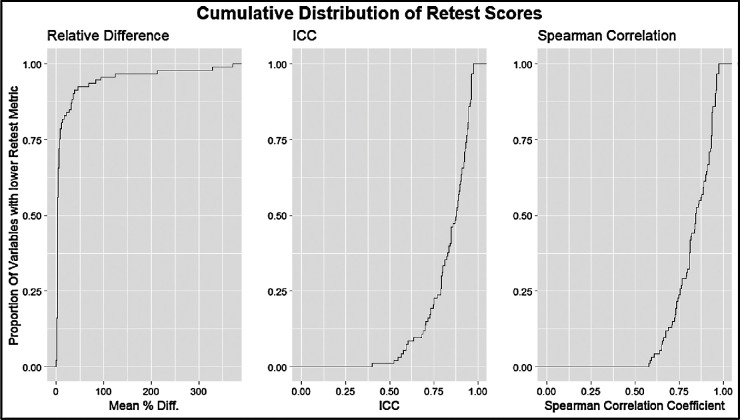
Cumulative distribution curves of retest quality metrics for the intravisit group. The *y*-axis represents the proportion of measured variables with lower retest. The *x*-axis from left to right shows the mean percentage difference between test and retest metrics of two-way, single-unit agreement ICC and Pearson correlation coefficient.

**Figure 8.**
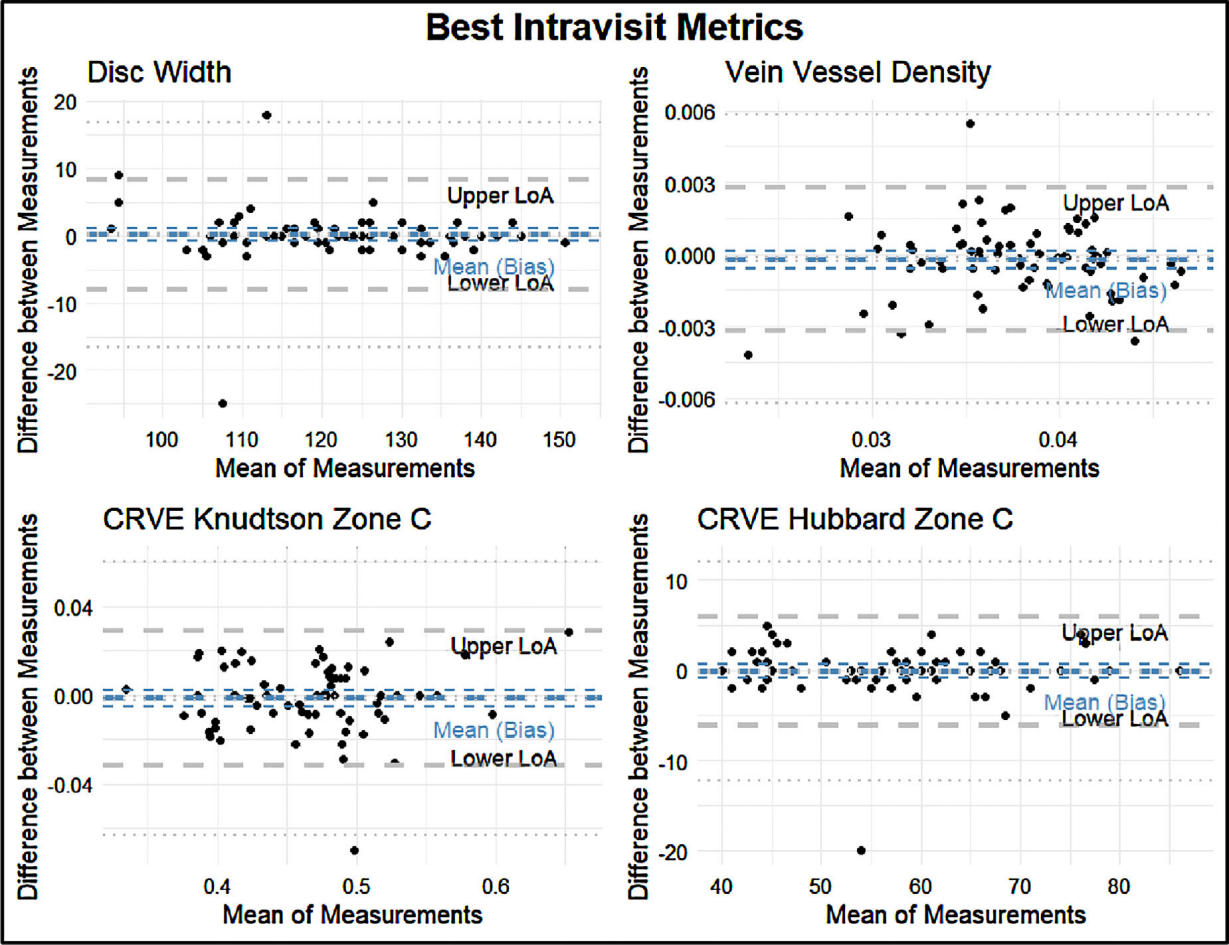
Bland–Altman plots of the four best metrics by Spearman correlation: disc width, vein vessel density, CRVE–Knudtson of Zone C, and CRVE–Hubbard of Zone C. The *thick blue line* indicates the means (bias) of the measurements. The *thick gray* *lines* indicate the upper and lower LoA. The *thin lines* indicate the 95% CIs for the means (bias) and LoA.

**Figure 9.**
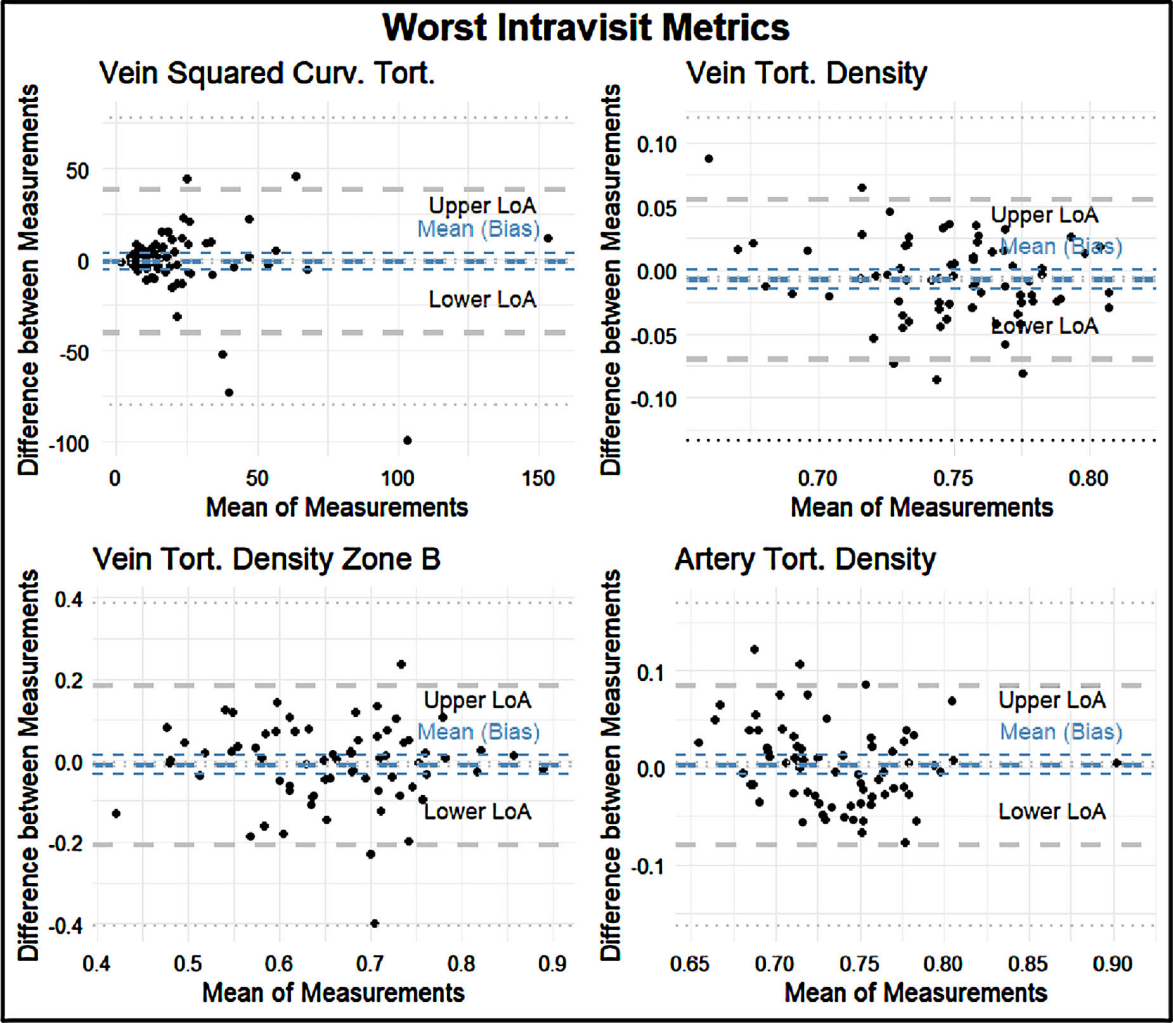
Bland–Altman plots of the four worst metrics in terms of retest reliability by Spearman correlation: artery distance tortuosity, vein tortuosity density, vein tortuosity density of Zone B, and artery tortuosity density. The plots feature the following: The *thick blue line* indicates the means (bias) of the measurements. The *thick gray lines* indicate the upper and lower LoA. The *thin lines* indicate the 95% CIs for the means (bias) and LoA.

The intravisit group had a mean percent difference range of 0.49% to 371.23%, an ICC range of 0.40 to 0.97, and a Spearman correlation coefficient of 0.55 to 0.96 (Fig. 7). The four variables with the best retest in the intravisit group with regard to the Spearman coefficient were disc width, vein vessel density, central retinal artery vein equivalent (CRVE)–Knudtson, and Hubbard in Zone C. The four variables with the worst retest reliability in terms of the Spearman correlation coefficient in the intravisit group were vein squared curvature tortuosity, vein tortuosity density, vein tortuosity density of Zone B, and artery tortuosity density (Figs. 8, 9; Table 5).

**Table 5.** Lower LoA, Mean LoA, and Upper LoA for each Bland–Altman Plot With a Precision of Two Significant Figures

	LoA		
Metric	Lower	Mean	Upper
Best intravisit metrics			
Disc width	−8.9	−0.23	8.4
Vein vessel density	−0.0029	−0.00010	0.0027
CRVE–Knudtson Zone C	−0.030	0.00	0.031
CRVE–Hubbard Zone C	−5.6	0.23	6.1
Worst intravisit metrics			
Vein squared curvature tortuosity	−33	2.7	38
Vein tortuosity density	−0.067	−0.010	0.056
Vein tortuosity density Zone B	−0.20	0.00	0.20
Artery tortuosity density	−0.085	0.00	0.085

**Figure 10.**
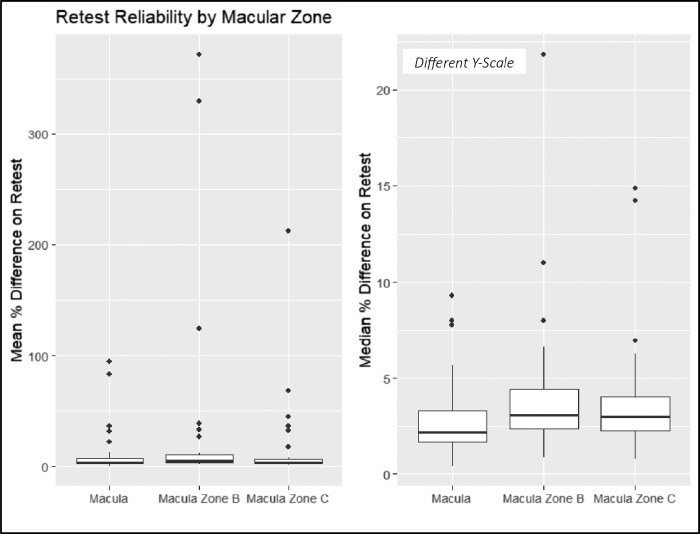
Mean (*left*) and median (*right*) percentage differences by zone for the intravisit group. Note the different *y*-scales of the box plots.

For the intravisit group, the mean percent difference on retest ranged from 0.49% to 94.71% in the entire macula, from 1.67% to 371.29% in Zone B, and from 1.35% to 212.42% in Zone C. The median retest ranged from 0.37% to 9.54%) for the entire macula, from 0.82% to 22.02% in Zone B, and from 0.81% to 15.23% in Zone C (Fig. 10).

Using a one-sided rank-sum test, we showed that, for the intervisit group, retest reliability in Zone C was better than that of Zone B (*P* = 0.007), and retest of Zone B was better than that of the entire macula (*P* = 0.035). These results did not translate to the intravisit group, in which retest quality between zones was not statistically significant when assuming a significance level of 0.05.

**Figure 11.**
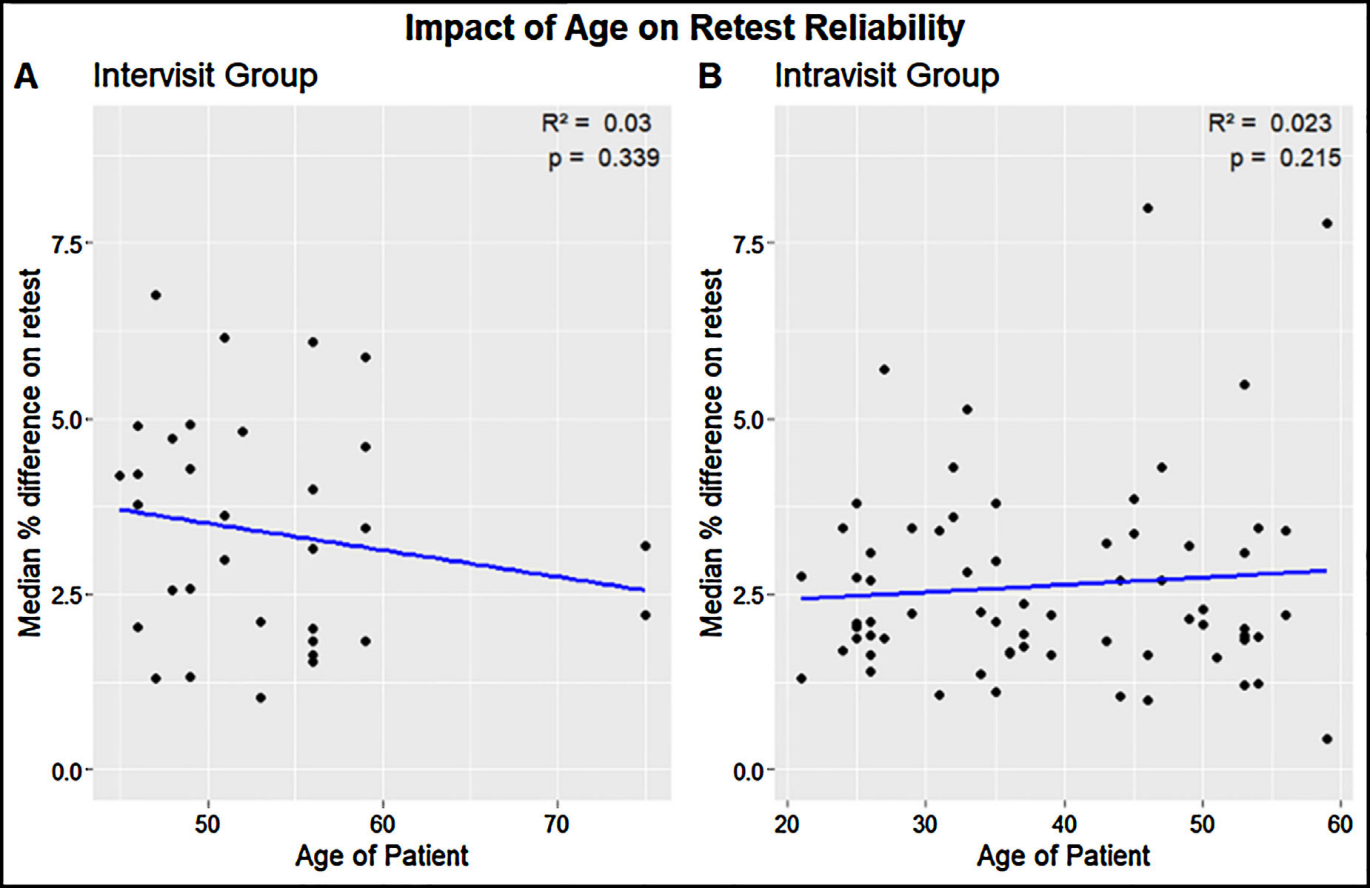
Age of patients versus the median percent on retest per subject examined. (*Left*) The intervisit group subjects with an *R*^2^ of 0.03 and *P* = 0.339. (*Right*) The intravisit group subjects with an *R*^2^ of 0.023 and *P* = 0.215.

A paired, two-sided Wilcoxon rank-sum test showed that the intravisit group had a better overall retest in terms of Spearman correlation coefficient than the intervisit group (*P* = 0.001). Using the same test, we could not find a statistically significant influence between gender and median retest reliability per subject (*P* = 0.45 for the intervisit group; *P* = 0.40 for the intravisit group). Likewise, using linear regression analysis, we could not find a statistically significant influence of age on median retest reliability per subject, with *R*^2^ = 0.03 and *P* = 0.339 for the intervisit group and *R*^2^ = 0.023 and *P* = 0.215 for the intravisit group (Fig. 11).

### Image Quality and Retest Reliability

**Figure 12.**
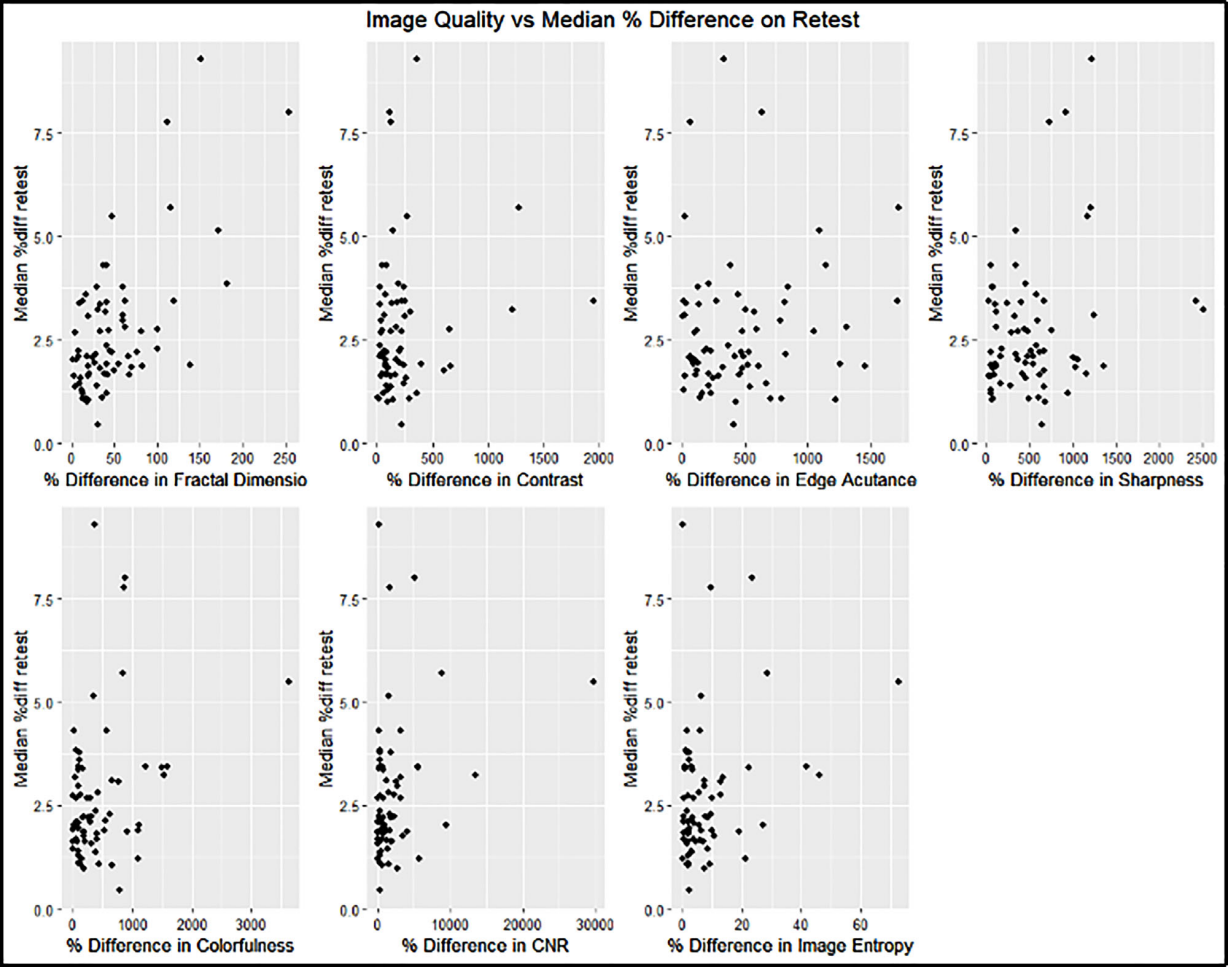
Influence of image quality on median retest reliability for the intravisit group. The *x*-axis indicates the percentage difference of the image quality variables measured for two intravisit images. The *y*-axis represents the median percentage difference of all measured metrics per image on retest.

In the intravisit group, all metrics except contrast and edge acutance showed a statistically significant correlation to the median retest per image of the intervisit group; however, no singular metric guaranteed good median retest reliability per image. The fractal dimension had the highest correlation to the median percent difference in retest (*R*^2^ = 0.40) (Fig. 12, Table 6).

**Table 6.** Correlation and Significance of Image Quality on Median Retest Reliability: Intravisit Group

Image Quality Metric	*R* ^2^	*P*
Fractal dimension	0.40	<0.001
Contrast	0.03	0.13
Edge acutance	0.01	0.38
Sharpness	0.07	0.03
Colorfulness	0.09	0.01
Contrast-to-noise ratio	0.08	0.02
Image entropy	0.09	0.01

**Figure 13.**
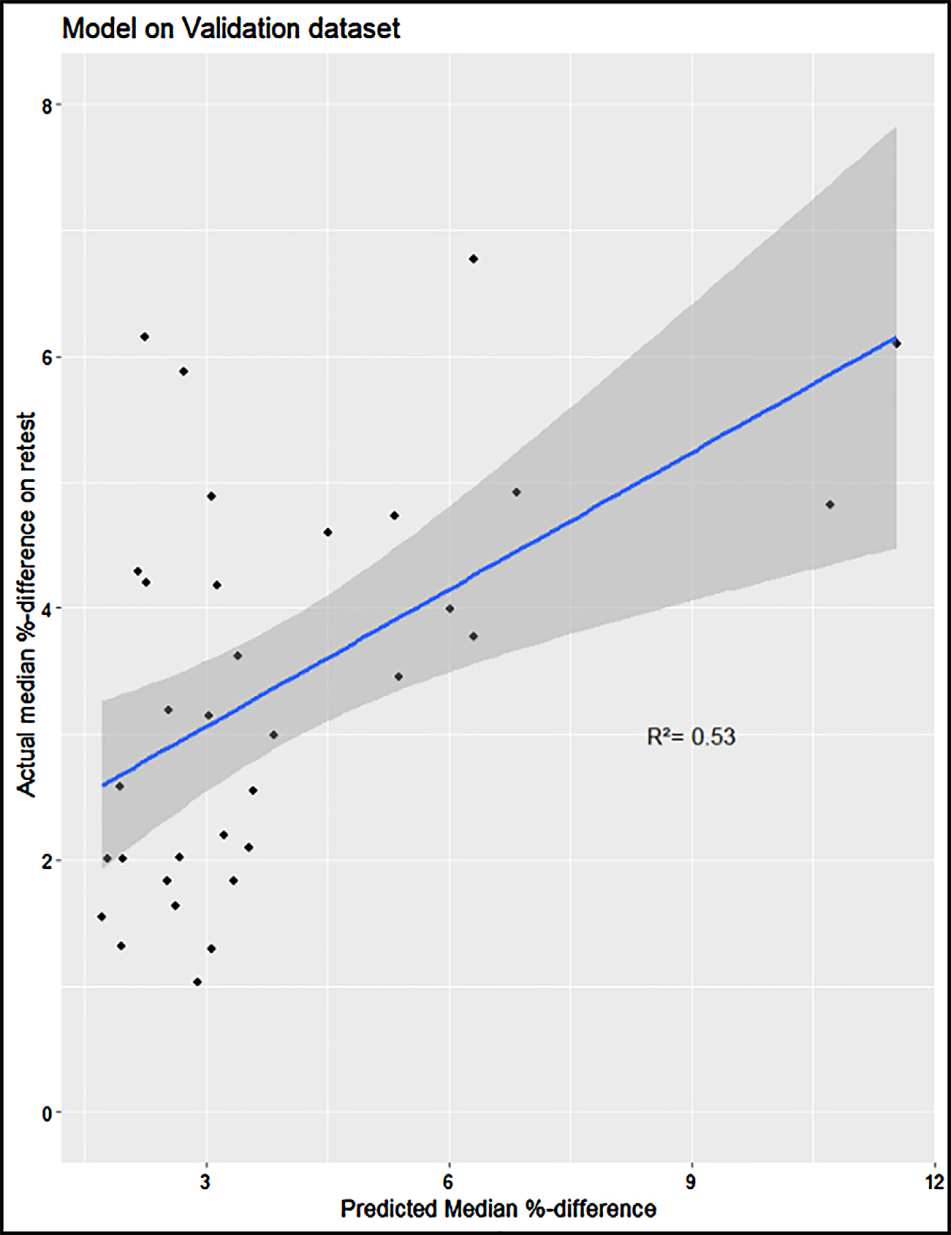
Constructed linear model on the intervisit validation set.

**Figure 14.**
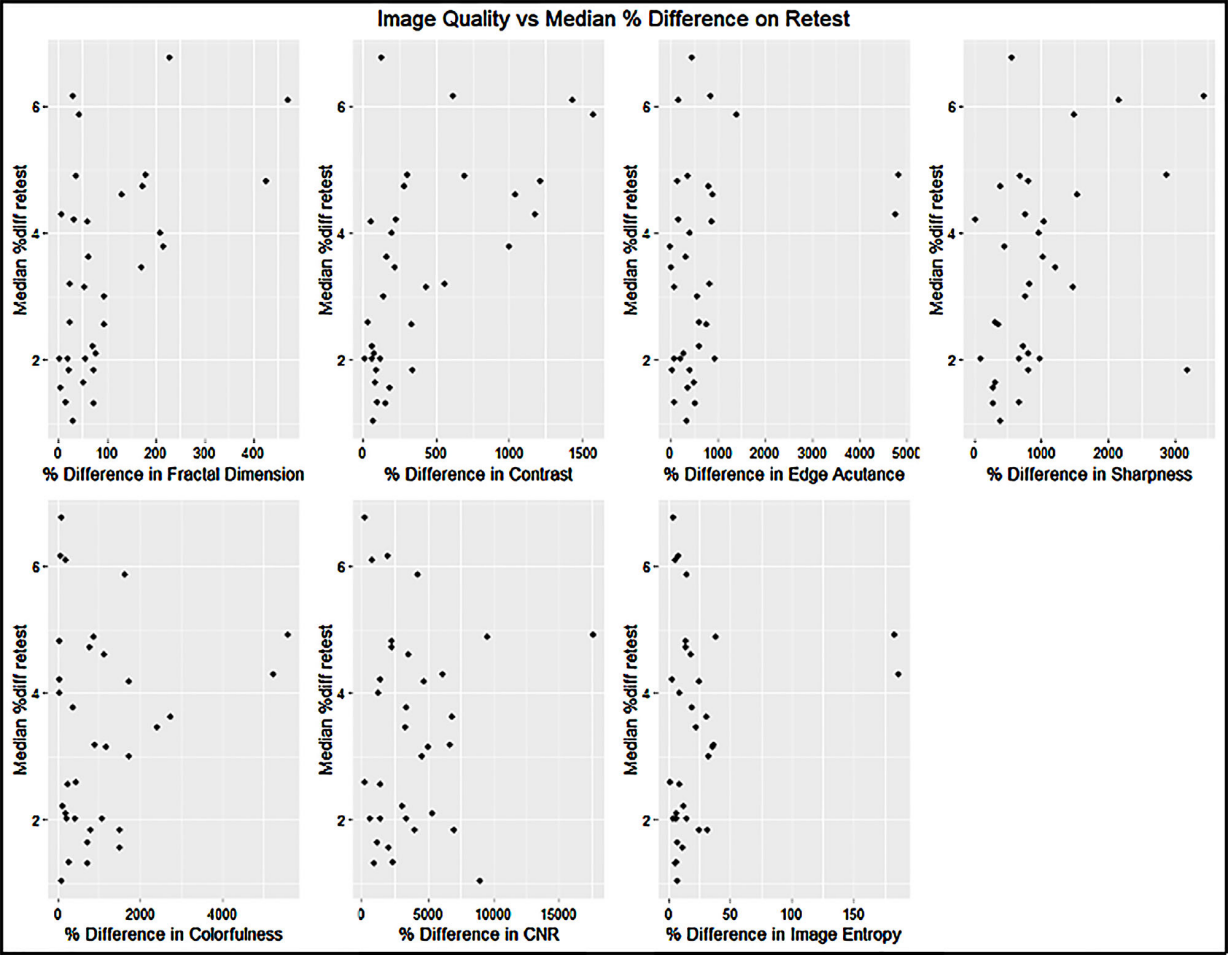
Influence of image quality on median retest reliability for the intervisit group.

As described in the Methods section, we constructed a linear model of the image quality metrics that were statistically significantly correlated to the median percentage difference in retest. The input variables are, therefore, fractal dimension, sharpness, colorfulness, contrast-to-noise ratio, and image entropy. Following a *P*-value–based backward selection process until our variance inflation factor fell below 5, we ended up with the remaining independent variables of fractal dimension and contrast-to-noise ratio. On the validation set, the model performed with an *R*^2^ = 0.53 (95% confidence interval [CI], 0.34–0.74), and a Pearson correlation test showed strong statistical significance (*P* < 0.0001). The linear model had a VIF < 1.1 (Fig. 13). In the intervisit group, the metrics of edge acutance, colorfulness, contrast-to-noise ratio, and image entropy did not show a statistically significant correlation to the median retest per image of the intervisit group. Contrast had the highest correlation to median percent difference in retest (*R*^2^ = 0.37), followed by fractal dimension (*R*^2^ = 0.27) (Fig. 14, Table 7).

**Table 7.** Correlation and Significance of Image Quality on Median Retest Reliability: Intervisit Group

Image Quality Metric	*R* ^2^	*P*
Fractal dimension	0.27	0.002
Contrast	0.37	<0.001
Edge acutance	0.07	0.13
Sharpness	0.15	0.02
Colorfulness	0.02	0.40
Contrast-to-noise ratio	0.03	0.73
Image entropy	0.04	0.25

## Discussion

We found that some biomarkers have exceptionally good test–retest reliability, consistent even among different cohorts and inclusion/exclusion criteria in healthy subjects. However, vessel characteristics under intense research, such as tortuosity and fractal dimension,^2^^,^^17^^,^^25^^,^^26^^,^^29^^–^^31^ have emerged as highly volatile biomarkers in both intervisit and intravisit datasets. AutoMorph, components of its pipeline, and retinal vessel segmentation algorithms have been used extensively in recent research to predict the risk of major cardiovascular events with an AUC of 0.78 (95% CI, 0.78–0.78),^27^^,^^33^ to characterize fundus photography changes for patients with schizophrenia,^17^ and, to predict the onset of Parkinson's disease with an AUC of 0.67 (95% CI, 0.65–0.69).^27^

The comprehensive evaluation of retinal parameters extracted from fundus photography stands as a crucial contribution to the realm of artificial intelligence–based biomarkers. The findings underscore the substantial variability in reliability across these parameters, indicating their inconsistent performance. Notably, developing a predictive model based on image quality metrics suggests a potential avenue for anticipating retest reliability, which could profoundly impact the reliability and applicability of artificial intelligence–driven analyses in ophthalmology. Moreover, considering the limited research on the reliability of vessel-segmentation algorithms, our study emphasizes the necessity of establishing more stable and consistent biomarkers. Enhanced reliability in these biomarkers could significantly impact clinical predictive analytics, potentially revolutionizing early disease detection and monitoring strategies for various conditions, ranging from cardiovascular risks to neurodegenerative diseases, thereby enhancing proactive healthcare interventions and opening the possibility of discovering associations strong enough to enable personalized treatment decision-making.

Examining pathways that lead to better image quality is a possible road to improvement. However, in this study, we found only a moderate correlation between the reliability of biomarkers and image quality, raising the question of whether future exploration of more retestable biomarkers is warranted. Finally, improving segmentation retest reliability will likely translate into improved retest-stable biomarkers; however, AutoMorph already represents a relatively high-quality segmentation tool with an AUC of up to 0.98, and there is limited room for future improvement.

Second, we collected 76 metrics from each fundus image; however, much of the observed variability stemmed from separating these metrics into the zones of the entire macula, Zone B, and Zone C. Although research has already been conducted for variables of the whole macula, variables for Zones B and C are less well understood. Furthermore, there are different calculation methods for the same metric. For example, there are myriad papers on calculating vessel tortuosity,^18^^–^^22^ and there exists more than one definitive way to calculate fractal dimension.^23^ Therefore, our results may not translate well to other calculation methods.

Third, imaging circumstances could have also played a role in our results. We found that retest reliability in the intravisit group was superior to that of the intervisit group (*P* = 0.001), and we found a statistically significant difference in the retest quality of zones in the intervisit group (*P* = 0.007 between Zone B and the macula; *P* = 0.035 between Zone C and the macula), which disappeared in the intravisit group. From this, we suggest that the current standards in fundus photography may still be improved. Further research should focus on which confounding variables are the most influential and how the confounders may be controlled. Our intravisit analysis group may provide a rough estimate of the optimal retest reliability attainable for each metric; however, new calculation methods of individual metrics may require a re-evaluation of repeatability.

Finally, it is possible that image quality influenced retest reliability. A statistically significant correlation existed between the examined image quality metrics and the median percent difference on retest per image. A linear contrast-to-noise ratio and fractal dimension difference model could achieve an *R*^2^ = 0.53 on its validation dataset; however, image quality could not predict the existence of outliers on select images on retest. These results demonstrate that merely improving the image quality will have only a moderate influence on overall retest. However, further research is necessary to see how the retest reliability of individual metrics may be calculated and what combination of image characteristics and metrics is more susceptible to outliers.

One limitation of our study is the use of a single imaging device, the ZEISS VISUCAM Pro NM camera, for all retinal image acquisitions. Although AutoMorph has been tested and validated on images from this device, it is important to acknowledge that results may vary when using different imaging equipment. Differences in hardware, such as sensor resolution and optics, as well as variations in image acquisition protocols, could potentially influence the metrics derived by AutoMorph. Although AutoMorph has been designed to be robust across various datasets, the exclusive use of one device in this study might limit the generalizability of our findings to images captured by other systems. We would expect retest reliability to perform even worse when including the added variability of different devices. Understanding the robustness of vessel segmentation techniques in relation to different fundus camera devices would help further establish the consistency and reliability of the software across different imaging environments.

We would also like to point out that AutoMorph has two separate training datasets for vessel and optic nerve head segmentation tasks. The better retest reliability of optic nerve head metrics could be due to more extensive or consistent ground-truth generation in optic nerve head segmentation training datasets. Additionally, we suspect some calculation methods from the vessel segmentation maps were more prone to numeric instability, leading to poorer retest reliability. Further research is needed to quantify the individual metric sensitivity toward segmentation map change.

In conclusion, this study provides an overview of the stability of the metrics that may be derived from color fundus photography and highlights the need for research to improve their stability. It provides an explanation for inconsistent findings in the association of retinal biomarkers with systemic diseases such as schizophrenia or dementia.^17^^,^^28^^,^^32^ Given the volatile nature of some biomarkers, improving ocular metrics already known to be associated with a systemic disease, such as the link between vessel tortuosity or vessel width and risk of stroke,^3^ seems necessary to provide an ocular biomarker–based personalized risk assessment. More generally, the low retest reliability of some examined biomarkers could conceal a potentially much stronger correlation between the currently examined biomarkers and their corresponding diseases. Improving retest reliability could allow disease progression to be examined in much smaller datasets or even allow for an individualized treatment approach.

## Supplementary Material

Supplement 1
