# Supplementary material for: Evaluating the Impact of Retinal Vessel Segmentation Metrics on Retest Reliability in a Clinical Setting: A Comparative Analysis Using AutoMorph
Source: Invest Ophthalmol Vis Sci. 2024 Nov 14;65(13):24. doi: 10.1167/iovs.65.13.24 (PMC11572755; doi:10.1167/iovs.65.13.24)
Supplement: Supplement 1 [file iovs-65-13-24_s001.docx]

**Supplementary Table 1 - Retest Reliability: Intervisit Group**

| **Variable** | **Spearman** | **ICC** | **Mean %-diff. On Retest** |
| --- | --- | --- | --- |
| Disc width | 0.986 | 0.982 | 2.684 |
| Cup width | 0.985 | 0.987 | 1.442 |
| Cup height | 0.983 | 0.981 | 3.119 |
| Disc height | 0.972 | 0.980 | 2.156 |
| CDR horizontal | 0.953 | 0.947 | 2.692 |
| CDR vertical | 0.936 | 0.934 | 3.814 |
| Fractal dimension C | 0.912 | 0.931 | 1.386 |
| CRVE Hubbard B | 0.896 | 0.898 | 3.427 |
| CRVE Knudtson B | 0.896 | 0.898 | 3.611 |
| AVR Knudtson B | 0.890 | 0.886 | 5.448 |
| Vessel density C | 0.887 | 0.867 | 6.355 |
| AVR Hubbard B | 0.878 | 0.883 | 5.283 |
| Vessel density vein C | 0.872 | 0.947 | 4.853 |
| Tortuosity density B | 0.868 | 0.809 | 5.732 |
| Vessel density vein B | 0.865 | 0.881 | 6.875 |
| Fractal dimension artery C | 0.860 | 0.868 | 2.330 |
| AVR Hubbard C | 0.832 | 0.901 | 4.083 |
| Distance tortuosity C | 0.826 | 0.834 | 19.215 |
| Average width artery C | 0.818 | 0.856 | 4.391 |
| Fractal dimension vein C | 0.818 | 0.837 | 1.976 |
| CRAE Hubbard C | 0.817 | 0.878 | 3.694 |
| AVR Knudtson C | 0.813 | 0.905 | 4.137 |
| Average width artery B | 0.811 | 0.817 | 5.630 |
| CRAE Hubbard B | 0.809 | 0.815 | 4.796 |
| CRAE Knudtson B | 0.809 | 0.821 | 5.038 |
| CRVE Knudtson C | 0.809 | 0.856 | 3.163 |
| Average width vein B | 0.807 | 0.790 | 4.404 |
| Vessel density | 0.804 | 0.796 | 5.556 |
| Distance tortuosity artery B | 0.803 | 0.839 | 24.799 |
| Vessel density B | 0.801 | 0.816 | 6.882 |
| CRAE Knudtson C | 0.799 | 0.880 | 3.755 |
| Distance tortuosity vein B | 0.791 | 0.715 | 28.691 |
| Squared curvature tortuosity vein B | 0.791 | 0.802 | 115.315 |
| Distance tortuosity artery | 0.789 | 0.831 | 28.386 |
| CRVE Hubbard C | 0.788 | 0.850 | 3.076 |
| Distance tortuosity artery C | 0.772 | 0.752 | 36.824 |
| Squared curvature tortuosity C | 0.772 | 0.833 | 58.862 |
| Vessel density artery C | 0.765 | 0.855 | 7.936 |
| Squared curvature tortuosity artery B | 0.756 | 0.753 | 107.581 |
| Squared curvature tortuosity vein | 0.755 | 0.576 | 62.293 |
| Distance tortuosity | 0.744 | 0.786 | 13.085 |
| Distance tortuosity vein | 0.736 | 0.681 | 29.085 |
| Fractal dimension vein B | 0.727 | 0.729 | 2.442 |
| Squared curvature tortuosity artery | 0.718 | 0.676 | 68.505 |
| Squared curvature tortuosity artery C | 0.718 | 0.882 | 97.844 |
| Average width artery | 0.715 | 0.699 | 3.921 |
| Average width C | 0.704 | 0.570 | 3.921 |
| Squared curvature tortuosity B | 0.684 | 0.314 | 4.347 |
| Tortuosity density artery | 0.681 | 0.712 | 4.347 |
| Distance tortuosity B | 0.679 | 0.650 | 212.799 |
| Fractal dimension | 0.675 | 0.666 | 5.058 |
| Tortuosity density artery B | 0.671 | 0.686 | 35.141 |
| Tortuosity density Vein | 0.659 | 0.686 | 1.285 |
| Vessel density artery B | 0.657 | 0.763 | 1.285 |
| Average width vein C | 0.654 | 0.596 | 9.054 |
| Tortuosity density artery C | 0.635 | 0.599 | 2.588 |
| Average width B | 0.611 | 0.677 | 9.480 |
| Distance tortuosity vein C | 0.600 | 0.819 | 4.485 |
| Fractal dimension B | 0.597 | 0.643 | 7.693 |
| Fractal dimension artery B | 0.596 | 0.755 | 4.876 |
| Squared curvature tortuosity | 0.596 | 0.606 | 34.462 |
| Vessel density artery | 0.589 | 0.587 | 1.908 |
| Fractal dimension vein | 0.576 | 0.539 | 2.810 |
| Vessel density vein | 0.570 | 0.549 | 34.646 |
| Tortuosity density C | 0.566 | 0.589 | 8.871 |
| Average width vein | 0.565 | 0.455 | 0.956 |
| Tortuosity density vein B | 0.563 | 0.525 | 0.964 |
| Squared curvature tortuosity vein C | 0.554 | 0.792 | 223.670 |
| Fractal dimension artery | 0.503 | 0.538 | 4.538 |
| Tortuosity density vein C | 0.497 | 0.504 | 5.846 |
| Average width | 0.347 | 0.420 | 5.846 |
| Tortuosity density | 0.344 | 0.429 | 9.886 |

**Supplementary Table 2 Retest Reliability Intravisit Group**

| **Variable** | **Spearman** | **ICC** | **Mean %-Diff. On Retest** |
| --- | --- | --- | --- |
| Disc width | 0.961 | 0.945 | 0.501 |
| Vessel density vein | 0.958 | 0.964 | 2.259 |
| CRVE Knudtson C | 0.956 | 0.955 | 2.177 |
| CRVE Hubbard C | 0.954 | 0.958 | 2.079 |
| Cup height | 0.951 | 0.974 | 3.202 |
| Vessel density | 0.949 | 0.945 | 3.094 |
| Vessel density artery | 0.947 | 0.943 | 5.051 |
| CDR vertical | 0.945 | 0.922 | 3.204 |
| Cup width | 0.945 | 0.962 | 0.71 |
| CRAE Knudtson B | 0.943 | 0.940 | 3.526 |
| CDR horizontal | 0.940 | 0.963 | 2.450 |
| Vessel density artery B | 0.938 | 0.905 | 9.008 |
| CRAE Hubbard B | 0.936 | 0.941 | 3.355 |
| CRAE Hubbard C | 0.933 | 0.932 | 3.330 |
| CRAE Knudtson C | 0.930 | 0.927 | 3.481 |
| AVR Hubbard C | 0.928 | 0.936 | 3.074 |
| AVR Knudtson C | 0.924 | 0.930 | 3.403 |
| Average width artery | 0.912 | 0.919 | 2.488 |
| Vessel density B | 0.908 | 0.878 | 6.399 |
| CRVE Knudtson B | 0.906 | 0.906 | 3.118 |
| Disc height | 0.903 | 0.847 | 2.385 |
| Vessel density vein C | 0.903 | 0.935 | 5.750 |
| AVR Hubbard B | 0.900 | 0.879 | 4.281 |
| CRVE Hubbard B | 0.900 | 0.909 | 2.937 |
| Vessel density vein B | 0.897 | 0.900 | 6.440 |
| Average width artery B | 0.896 | 0.893 | 4.305 |
| Average width artery C | 0.887 | 0.897 | 3.594 |
| Fractal dimension C | 0.885 | 0.875 | 1.348 |
| Vessel density artery C | 0.885 | 0.904 | 8.362 |
| AVR Knudtson B | 0.880 | 0.793 | 4.985 |
| Fractal dimension | 0.875 | 0.889 | 0.492 |
| Vessel density C | 0.868 | 0.896 | 6.164 |
| Fractal dimension artery | 0.866 | 0.846 | 1.073 |
| Fractal dimension vein C | 0.843 | 0.841 | 1.972 |
| Fractal dimension vein | 0.835 | 0.884 | 0.752 |
| Fractal dimension artery C | 0.826 | 0.811 | 2.099 |
| Average width | 0.820 | 0.834 | 2.499 |
| Average width vein C | 0.820 | 0.800 | 3.631 |
| Tortuosity density | 0.809 | 0.811 | 1.996 |
| Fractal dimension artery B | 0.807 | 0.750 | 2.497 |
| Tortuosity density B | 0.800 | 0.752 | 7.965 |
| Average width B | 0.793 | 0.788 | 4.857 |
| Tortuosity density C | 0.785 | 0.823 | 3.724 |
| Squared curvature tortuosity | 0.783 | 0.860 | 31.556 |
| Average width vein | 0.772 | 0.791 | 3.321 |
| Average width C | 0.766 | 0.748 | 4.438 |
| Average width vein B | 0.766 | 0.545 | 5.009 |
| Fractal dimension B | 0.764 | 0.717 | 1.665 |
| Distance tortuosity B | 0.758 | 0.770 | 26.512 |
| Fractal dimension vein B | 0.757 | 0.791 | 2.358 |
| Distance tortuosity C | 0.754 | 0.733 | 17.298 |
| Distance tortuosity vein C | 0.753 | 0.603 | 35.868 |
| Distance tortuosity vein B | 0.750 | 0.726 | 38.589 |
| Squared curvature tortuosity vein B | 0.744 | 0.703 | 371.289 |
| Squared curvature tortuosity B | 0.743 | 0.799 | 238.104 |
| Distance tortuosity | 0.735 | 0.829 | 12.565 |
| Tortuosity density vein C | 0.728 | 0.691 | 6.930 |
| Distance tortuosity artery C | 0.707 | 0.732 | 32.131 |
| Distance tortuosity artery | 0.682 | 0.523 | 36.138 |
| Squared curvature tortuosity C | 0.681 | 0.882 | 45.089 |
| Squared curvature tortuosity artery | 0.673 | 0.401 | 94.586 |
| Distance tortuosity artery B | 0.670 | 0.790 | 32.719 |
| Distance tortuosity vein | 0.659 | 0.796 | 22.122 |
| Squared curvature tortuosity artery B | 0.643 | 0.869 | 329.388 |
| Tortuosity density artery C | 0.632 | 0.701 | 6.448 |
| Squared curvature tortuosity artery C | 0.626 | 0.681 | 67.964 |
| Tortuosity density artery B | 0.624 | 0.633 | 222.891 |
| Squared curvature tortuosity vein C | 0.618 | 0.577 | 212.419 |
| Squared curvature tortuosity vein | 0.568 | 0.701 | 82.735 |
| Tortuosity density vein | 0.566 | 0.591 | 3.498 |
| Tortuosity density artery | 0.552 | 0.591 | 94.712 |
| Tortuosity density vein B | 0.552 | 0.567 | 11.847 |
